# Motor Areas Show Altered Dendritic Structure in an Amyotrophic Lateral Sclerosis Mouse Model

**DOI:** 10.3389/fnins.2017.00609

**Published:** 2017-11-01

**Authors:** Matthew J. Fogarty, Erica W. H. Mu, Nickolas A. Lavidis, Peter G. Noakes, Mark C. Bellingham

**Affiliations:** ^1^Faculty of Medicine, School of Biomedical Sciences, University of Queensland, St Lucia, QLD, Australia; ^2^Queensland Brain Institute, University of Queensland, St Lucia, QLD, Australia

**Keywords:** dendrite, spine density, striatum, hippocampus, hypoglossal motor neuron, trochlear motor neuron, lumbar motor neuron

## Abstract

**Objective:** Motor neurons (MNs) die in amyotrophic lateral sclerosis (ALS), a clinically heterogeneous neurodegenerative disease of unknown etiology. In human or rodent studies, MN loss is preceded by increased excitability. As increased neuronal excitability correlates with structural changes in dendritic arbors and spines, we have examined longitudinal changes in dendritic structure in vulnerable neuron populations in a mouse model of familial ALS.

**Methods:** We used a modified Golgi-Cox staining method to determine the progressive changes in dendritic structure of hippocampal CA1 pyramidal neurons, striatal medium spiny neurons, and resistant (trochlear, IV) or susceptible (hypoglossal, XII; lumbar) MNs from brainstem and spinal cord of mice over-expressing the human SOD1^G93A^ (SOD1) mutation, in comparison to wild-type (WT) mice, at four postnatal (P) ages of 8–15, 28–35, 65–75, and 120 days.

**Results:** In SOD1 mice, dendritic changes occur at pre-symptomatic ages in both XII and spinal cord lumbar MNs. Spine loss without dendritic changes was present in striatal neurons from disease onset. Spine density increases were present at all ages studied in SOD1 XII MNs. Spine density increased in neonatal lumbar MNs, before decreasing to control levels by P28-35 and was decreased by P120. SOD1 XII MNs and lumbar MNs, but not trochlear MNs showed vacuolization from the same time-points. Trochlear MN dendrites were unchanged.

**Interpretation:** Dendritic structure and spine alterations correlate with the neuro-motor phenotype in ALS and with cognitive and extra-motor symptoms seen in patients. Prominent early changes in dendritic arbors and spines occur in susceptible cranial and spinal cord MNs, but are absent in MNs resistant to loss in ALS.

## Introduction

In amyotrophic lateral sclerosis (ALS) both upper and lower motor neurons (MNs), as well as the corticospinal tract, undergo progressive degeneration (Charcot and Joffroy, 1869; Cleveland and Rothstein, 2001). This loss of MNs in the brainstem and spinal cord inevitably causes worsening muscular atrophy and weakness, typically causing death 3–5 years following diagnosis (Cleveland and Rothstein, 2001; Hardiman et al., 2011), usually from respiratory complication or failure (Connolly et al., 2015). The Golgi-Cox technique has demonstrated significant structural changes, including dendritic shortening and decreased spine density, in upper and lower motor neurons of ALS patients and animal models of familial ALS (Hammer et al., 1979; Horoupian et al., 1984; Udaka et al., 1986; Kato et al., 1987; Fogarty et al., 2016c). These structural abnormalities are correlated with cortical hyper-excitability (Eisen et al., 1993; Mills and Nithi, 1997; Zanette et al., 2002; Turner et al., 2005; Vucic and Kiernan, 2006; Fogarty et al., 2015, 2016b), which can be observed before diagnosis in certain ALS patients (Vucic et al., 2008), suggesting that protracted preclinical structure/function alterations play a key role in disease pathogenesis (van Zundert et al., 2012; Eisen et al., 2014).

The dendritic structure of the neuron integrates the diverse stream of excitatory, inhibitory and neuromodulatory synaptic inputs, which are the key determinants of whether an action potential is generated (Luebke et al., 2010). Regulation of dendrite length and the density of dendritic spines normally occurs during brain development and aging, in addition to many psychiatric or neurodegenerative pathologies, and these changes are correlated with alteration in neurotransmitter activity (Luebke et al., 2010; Sala and Segal, 2014). In particular, glutamatergic neurotransmission is thought to be a significant regulator of dendritic structure, and glutamatergic excitotoxicity is one proposed mechanism in ALS pathogenesis (Eisen et al., 1993; Mills and Nithi, 1997; van Zundert et al., 2008, 2012; Bogaert et al., 2010; Bellingham, 2011; Turner et al., 2013; Devlin et al., 2015; Fogarty et al., 2015, 2016b; Saba et al., 2015).

The hSOD1^G93A^ transgenic mouse (SOD1) is the most prevalent animal model of ALS, overexpressing the human SOD1G93A mutation (Gurney et al., 1994; Turner and Talbot, 2008). SOD1 mutations are found in 10–20% of familial ALS and in 1–2% of sporadic ALS cases, thus accounting for about 2% of all ALS cases (Taylor et al., 2016). This SOD1 mouse model of familial ALS shows significant alterations in dendritic structure, including the density of dendritic spines, of primary motor cortex upper MNs (Spalloni et al., 2011; Jara et al., 2012; Fogarty et al., 2015, 2016b; Saba et al., 2015), pyramidal neurons of medial pre-frontal cortex (MPFC) (Sgobio et al., 2008; Fogarty et al., 2016c), and lower MNs in the brainstem and spinal cord (van Zundert et al., 2008; Martin et al., 2013). Although these studies have provided insights into individual components of the neuro-motor network at specific time points in disease pathogenesis, there remains a need to define the onset and progression of neuronal pathology at successive stages of disease in ALS in key subcortical structures receiving cortical outputs: the striatum, hippocampus (Spalloni and Longone, 2015), brainstem, and spinal cord.

Our results show that dendritic atrophy and spine loss in striatal medium spiny neurons (MSNs) and lower MNs of lumbar spinal cord contrast starkly with our observations of increased dendritic arbor and spine density in brainstem hypoglossal (XII) MNs and unchanged morphology of trochlear (IV) MNs, a brainstem lower MN population which is resistance to degeneration in the SOD1 mouse (Ferrucci et al., 2010). Dendritic vacuolisation of SOD1 XII MNs and lumbar MNs occurred from the same time-point, while IV MNs did not show significant vacuolization or dendritic changes at any time point studied. These data may hint at independent mechanisms of degeneration between these MN populations and other upper and lower MNs. In addition to dendritic changes in the lower MN populations severely affected by ALS (Hammer et al., 1979; Horoupian et al., 1984; Udaka et al., 1986; Kato et al., 1987; Cleveland and Rothstein, 2001; Aggarwal and Nicholson, 2002; Hardiman et al., 2011; Turner et al., 2013; Eisen et al., 2014), we also observed hitherto unreported degenerative changes in neurons within the dorsal striatum.

## Materials and methods

### Ethics statement

Fifty two age- and litter-matched wild-type (WT) and heterozygous transgenic mice overexpressing the hSOD1^G93A^ mutation were utilized. This study was conducted following the guidelines of the Queensland Government Animal Research Act 2001, associated Animal Care and Protection Regulations (2002 and 2008), as well as the Australian Code for the Care and Use of Animals for Scientific Purposes, 8th Edition (National Health and Medical Research Council, 2013). The University of Queensland Anatomical Biosciences Animal Ethics Committee approved all protocols involving animal use.

### Golgi-Cox impregnation and processing

Mice from four previously characterized phenotypic stages were used—(1) postnatal (P) days P8-15 (7 WT and 5 SOD1 mice), when lower MNs show early signs of intrinsic and synaptic hyper-excitability (van Zundert et al., 2008; Fogarty et al., 2015); (2) P28-25 (5 WT and 5 SOD1 mice), pre-symptomatic stage when mice do not show discernable symptoms but do show synaptic and structural changes in neurons (Sgobio et al., 2008; van Zundert et al., 2008; Ozdinler et al., 2011; Fogarty et al., 2015; Saba et al., 2015); (3) P65-75 (5 WT and 5 SOD1 mice), representing disease onset, with the earliest period of MN loss, muscle weakness, and appearance of overt symptoms (Ozdinler et al., 2011; Jara et al., 2012; Lee et al., 2013; Steyn et al., 2013) and; (4) P110-130 (8 WT and 12 SOD1 mice, a mid-disease stage where mice show marked muscle mass and loss of muscle strength, respectively (Lee et al., 2013). In our SOD1 colony, end stage disease (loss of righting reflex) occurs between 150 and 175 days (Ngo et al., 2012).

Mice were anesthetized with sodium pentobarbitone (60–80 mg/kg intraperitoneally, Vetcare) and then a heparinized needle (Sigma-Aldrich) was used to intracardially exsanguinate the animal. Whole brain, brainstem, and lumbar spinal cord tissues were incubated for 5 days at 37°C in the dark in a modified rapid Golgi-Cox solution that contained 5% potassium dichromate, 5% potassium chromate, 5% mercuric chloride, as described previously (Rutledge et al., 1969) (all chemicals Sigma-Aldrich).

Brain slices (300 μm thickness) were cut with a vibrating Zeiss Hyrax V50 microtome (Carl Zeiss) to produce off-coronal (15° rostral rotation) forebrain sections, transverse brainstem sections, and parasagittal lumbar spinal cord sections (embedded in 10% agarose block in 0.1 M phosphate buffered saline [PBS]). Serial sections in 24-well plates were sequentially incubated in 30% sucrose in 0.1 M PBS (30 min), 50% ethanol (dehydration, 5 min), 0.1 M ammonium hydroxide solution (30 min, rinsed twice in distilled water (5 min), and then immersed in aluminum sulfate-based Fujihunt photo fixer (Fuijfilm, Singapore, 30 min) while protected from light exposure. After rinsing in distilled water twice (5 min each), sections were dehydrated in ethanol (70, 90, 95, and three rinses of 100%, 5 min each) and transferred to chloroform: xylene: alcohol (CXA) solution (1:1:1 ratio, 10 min). Sections were cleared in xylene (2 × 5 min each) and mounted using DPX (Sigma-Aldrich) on Superfrost Plus (Lomb Menzel Glaser) slides. Slides were air dried and stored in the dark. We note that aged mice brain tissue required longer xylene (extra 5 min) clearing due to a higher fat content.

### Region selection

Dorsal striatum neurons for tracing were selected from sections between bregma ~1.9 and 0.6 mm, within the striatum, which was located lateral to the lateral ventricle and ventral and medial to the corpus callosum. Hippocampal CA1 pyramidal neurons for tracing were selected from sections between bregma ~−1.1 and −2.6 mm. A mouse brain atlas (Franklin and Paxinos, 2008) was used to help define the boundaries of these areas.

### Neuronal tracing

Morphological properties (dendritic branching, length and dendritic spines) of Golgi-impregnated neurons were traced with a 63x objective (NA 1.4) of an Axioskop 2 microscope (Carl Zeiss) with an automated *z*-stage controlled by Neurolucida™ software (MBF Bioscience Inc.) as for previous reports (Fogarty et al., 2015, 2016c; Kanjhan et al., 2015). We classified small processes as spines only if they met criteria of <3 μm length and <0.8 μm cross-sectional diameter (Harris, 1999; Fogarty et al., 2015). These tracings yielded: (i) soma (irregular ellipsoid) volume (based on cross-sectional areas as previously; Kanjhan et al., 2015); (ii) total arbor length (the summed dendritic arbor length); (iii) apical or basal length (summed single apical tree length or summed length of all basal trees); (iv) mean basal tree or mean dendritic length (the mean length of each basal or dendritic tree from the soma); (v) apical and basal reach (farthest linear distance between apical or basal dendrite ending and soma); (vi) apical and basal ramifications (number of bifurcations before dendritic termination, a measure of neuronal complexity); and (vii) apical or basal dendritic spine density (number of dendritic spines per 100 μm of dendrite), as defined in our previous study (Fogarty et al., 2015; Kanjhan et al., 2015). In total, 1.34 m of dendrite length were traced in the 404 neurons that were used in this study.

All analyzed pyramidal neurons had a minimum of three intact basal dendritic trees and an apical dendrite that reached dendritic termination without exiting the brain section (Klenowski et al., 2015). Dendritic spines were assessed along the entire apical or basal dendritic arbor, as for previous studies (Fogarty et al., 2016c). Striatal MSNs were identified as for previous studies (Rafols and Fox, 1979), with spine densities of >10 spines/μm (Klenowski et al., 2016) required for inclusion, as aspiny neurons with similar shaped dendritic trees occur in rodent striatum (Rafols and Fox, 1979). The IV and XII motor nucleus were identified with a mouse brain atlas, relative to major landmarks including the aqueduct, medial longitudinal fasciculus and central canal (Franklin and Paxinos, 2008), with MNs selected by their large soma and multipolar dendrites. Lumbar MNs were identified as large neurons with longitudinal soma lengths >25 μm and dendrites arranged rostro-caudally, as for past studies (Bellinger and Anderson, 1987).

### Imaging

Photomicrographs of dendritic arbors were generated from minimum intensity *z*-stack projections (*z*-step size of 2 μm between images; stack depth between 20 and 100 images) that were joined digitally to form mosaics of complete cells. The imaged area was estimated from the overlaid tracing file. The mosaics appear “tessellated” because of different individual *z*-stack thicknesses. Representative images of spine density were taken with a 100x objective (Carl Zeiss) image at a single focal plane.

### Statistical methods

Prism 6 (Graphpad) was used for all statistical analysis. Data are given as mean ± SEM and represent the mean of all single neurons from an individual brain, brainstem or spinal cord region per mouse i.e., the *n* used for statistical purposes was an individual animal as the independent variable, as consistent with previous reports using the Golgi technique (Fogarty et al., 2016c). For the striatum, CA1, and brainstem regions, a minimum of two thick sections was used. The number of individual neuronal observations that contributed to these summary data is reported in the relevant tables, with *a priori* exclusion criteria, namely, individual data points from neurons that were beyond two standard deviations from the mean. Data was assessed for normality using a D'Agostino and Pearson omnibus test. Two-way ANOVAs with Bonferroni's post-test were applied to data with significant differences due to genotype were accepted as statistically significant with adjusted *P* (adj. *P*) values ^*^P < 0.05, ^**^P < 0.01, ^***^P < 0.001, and ^****^P < 0.0001. Percentage changes are reported in relation to the WT mean for significantly different age groups. The researcher performing tracings (MJF) was blind to genotype.

## Results

### Spine density loss occurs from P65-75 onward in striatum MSNs from SOD1 mice

**Figure 1:**
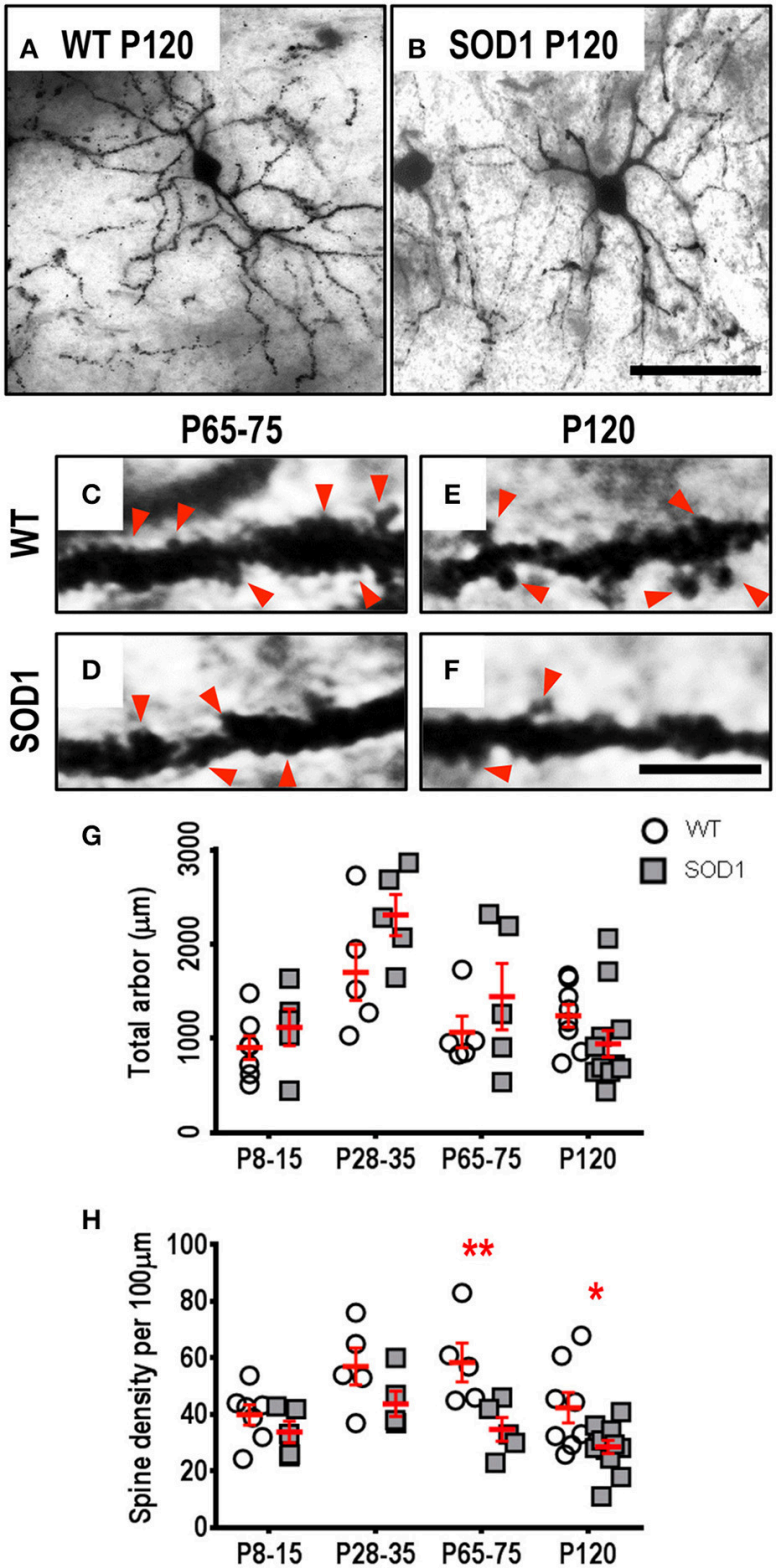
Decreased dendritic spine density of MSNs within the striatum commencing from P65-75 in SOD1 mice, compared to WT controls. Images show mosaics of striatum MSNs from P120 WT **(A)** and SOD1 **(B)** mice, note our representative image represents the mean MSN dendritic length in 7 of 12 P120 SOD1 mice was decreased compared to the lowest WT value at mid-disease ages. High-magnification images of dendrites from P65-75 WT **(C)**, SOD1 **(D)**, and P120 WT **(E)**, and SOD1 **(F)** mice MSNs are also shown, with examples of some of the dendritic spines identified using red arrows, representative of the spiny structures and not an exhaustive identification of all quantified spines on these images. **(G)** shows a scatterplot quantifying unchanged total dendritic arbor length (μm) of SOD1 MSNs (gray squared), compared to WT controls (white circles). **(H)** shows a scatterplot quantifying significantly decreased dendritic spine density per 100 μm dendrite of SOD1 MSNs (gray squared) compared to WT controls (white circles) at P65-75 and P120. All data mean ± SEM, with two-way ANOVAs followed by Bonferroni post-tests, *P < 0.05 and **P < 0.01. *n* = 7, 5, 5, and 8 for WT P8-15, P28-35, P65-75, and P120 respectively. *n* = 6, 5, 5, and 12 for SOD1 P8-15, P28-35, P65-75, and P120 respectively. Scale bar: **(A,B)**, 75 μm. Dendritic frame width **(C–F)**, 20 μm.

The soma volume, total arbor length, mean tree length, dendritic reach or dendritic ramifications of striatum MSNs in SOD1 mice did not differ from WT MSNs at any age (Table 1, Figure 1). By contrast, SOD1 MSNs showed significantly decreased dendritic spine density from P65-75 onward (Figures 1C–F,H). While dendritic spine density of SOD1 and WT striatal MSNs was not significantly different at P8-16 or P28-35 (Table 1, Figure 1H), the dendritic spine density of SOD1 MSNs was reduced by 40% at P65-75 (adj. *P* = 0.0082^**^, Table 1, Figure 1H), and by 33% at P120 (adj. *P* = 0.043^*^, Table 1, Figure 1H).

**Table 1:** Morphometric dendritic and dendritic spine parameters of MSNs within the striatum.

**Region**	**P8-15 (*n*) Neonatal**	**P28-35 (*n*) Pre-symptomatic**	**P65-75 (*n*) Symptom Onset**	**P120 (*n*) Mid-disease**
Soma volume (μm^3^)	WT: 5932 ± 1122 (*7*) SOD1: 7065 ± 1937 (*5*) Not significant	WT: 10170 ± 1904 (*5*) SOD1: 5867 ± 698 (*5*) Not significant	WT: 7520 ± 1344 (*5*) SOD1: 7907 ± 1357 (*5*) Not significant	WT: 6039 ± 1218 (*8*) SOD1: 7763 ± 623 (*12*) Not significant
Total arbor length (μm)	WT: 904 ± 125 (*7*) SOD1: 1119 ± 195 (*5*) Not significant	WT: 1701 ± 298 (*5*) SOD1: 2309 ± 218 (*5*) Not significant	WT: 1068 ± 168 (*5*) SOD1: 1444 ± 351 (*5*) Not significant	WT: 1241 ± 121 (*8*) SOD1: 944 ± 139 (*12*) Not significant
Mean tree length (μm)	WT: 236 ± 30 (*7*) SOD1: 378 ± 79 (*5*) Not significant	WT: 379 ± 65 (*5*) SOD1: 485 ± 65 (*5*) Not significant	WT: 322 ± 47 (*5*) SOD1: 453 ± 94 (*5*) Not significant	WT: 335 ± 45 (*8*) SOD1: 249 ± 41 (*12*) Not significant
Dendritic reach (μm)	WT: 154 ± 19 (*7*) SOD1: 255 ± 51 (*5*) Not significant	WT: 296 ± 36 (*5*) SOD1: 321 ± 23 (*5*) Not significant	WT: 171 ± 20 (*5*) SOD1: 190 ± 24 (*5*) Not significant	WT: 227 ± 25 (*8*) SOD1: 157 ± 16 (*12*) Not significant
Dendritic ramifications	WT: 3.6 ± 0.4 (*7*) SOD1: 3.3 ± 0.3 (*5*) Not significant	WT: 3.8 ± 0.3 (*5*) SOD1: 4.6 ± 0.3 (*5*) Not significant	WT: 3.8 ± 0.4 (*5*) SOD1: 4.2 ± 0.5 (*5*) Not significant	WT: 4.5 ± 0.4 (*8*) SOD1: 4.6 ± 0.3 (*12*) Not significant
Dendritic spine density per 100 μm	WT: 40 ± 3.6 (*7*) SOD1: 33.8 ± 3.8 (*5*) Adj. *P* > 0.9999	WT: 57 ± 6.5 (*5*) SOD1: 43.8 ± 4.5 (*5*) Adj. *P* = 0.2936	WT: 58.4 ± 6.9 (*5*) SOD1: 34.8 ± 4.1 (*5*) Adj. *P* = 0.0082^**^	WT: 42.4 ± 5.4 (*8*) SOD1: 28.6 ± 2.3 (*12*) Adj. *P* = 0.0428^*^

All data presented as mean ± SEM. All analyses were unpaired two-way ANOVAs for age and genotype, comparing soma volume (Age: P = 0.6285; Genotype: P = 0.7725), total dendritic arbor length (Age: P < 0.0001^****^; Genotype: P = 0.1201), mean dendritic tree length (Age: P = 0.0575; Genotype: P = 0.0824), dendritic reach (Age: P = 0.0002^***^; Genotype: P = 0.4472), dendritic ramifications (Age: P = 0.0379^*^; Genotype: P = 0.6085), and dendritic spine density per 100 μm (Age: P = 0.0041^**^; Genotype: P < 0.0001^****^). Bonferroni post-tests were used to compare the effect of genotype at each age group, with the adjusted P-value (adj. P) reported in the table for parameters that had significant genotype effects. Each animal used (n) contains mean values from multiple individual neurons, specifically P8-15 (WT: 11 neurons; SOD1: 12 neurons), P28-35 (WT: 8 neurons; SOD1: 9 neurons), P65-75 (WT: 9 neurons; SOD1: 10 neurons), and P120 (WT: 12 neurons; SOD1: 16 neurons).

Spine loss in MSNs has not been reported in any ALS rodent model. Corticostriatal inputs from motor and prefrontal cortex are critical for striatal regulation of goal-directed movements (Anderson et al., 2010; Rothwell et al., 2015). TDP-43 positive-inclusions occur in the striatum of ALS patients (Zhang et al., 2008), and spine density loss and synaptic hyper-activity in the SOD1 mouse motor cortex precedes dendritic shrinkage (Jara et al., 2012; Yasvoina et al., 2013; Fogarty et al., 2015, 2016c). Thus, increased corticostriatal output may contribute to MSN synaptic dysfunction, which may be involved in extrapyramidal symptoms of ALS.

### SOD1 hippocampal CA1 pyramidal neurons show no morphological differences to WT neurons at any age

While morphological changes in motor and prefrontal cortex of SOD1 mice occurs well before any disease phenotype (Jara et al., 2012; Yasvoina et al., 2013; Fogarty et al., 2015, 2016c), entorhinal cortex pyramidal neurons of SOD1 mice are unaltered (Fogarty et al., 2016c). The hippocampal CA1 region is a major output target of the entorhinal cortex, which also processes inputs from multiple cortical areas (van Groen et al., 2003) and is associated with cognitive deficits in clinical ALS (Strong et al., 2009). We therefore measured the dendritic arbors and dendritic spine densities of hippocampal CA1 pyramidal neurons, and found that there were no differences in any morphological measurement of hippocampal CA1 pyramidal neurons of SOD1 mice compared to WT controls at any age (Table 2). This lack of hippocampal pathology in SOD1 mice accords with the rarity of cognitive dysfunction in ALS patients with SOD1 mutations (Wicks et al., 2009).

**Table 2:** Morphometric dendritic and dendritic spine parameters of pyramidal neurons within the hippocampus CA1.

**Region**	**P8-15 (*n*) Neonatal**	**P28-35 (*n*) Pre-symptomatic**	**P65-75 (*n*) Symptom Onset**	**P120 (*n*) Mid-disease**
Soma volume (μm^3^)	WT: 11379 ± 2056 (*7*) SOD1: 10426 ± 2207 (*5*) Not significant	WT: 9261 ± 805 (*5*) SOD1: 9207 ± 1472 (*5*) Not significant	WT: 17942 ± 3159 (*5*) SOD1: 20248 ± 5501 (*5*) Not significant	WT: 12867 ± 3509 (*6*) SOD1: 16239 ± 2480 (*7*) Not significant
Total arbor length (μm)	WT: 2821 ± 626 (*7*) SOD1: 1850 ± 476 (*5*) Not significant	WT: 2844 ± 213 (*5*) SOD1: 4058 ± 310 (*5*) Not significant	WT: 4442 ± 881 (*5*) SOD1: 3897 ± 209 (*5*) Not significant	WT: 3190 ± 351 (*6*) SOD1: 2970 ± 334 (*7*) Not significant
Apical arbor length (μm)	WT: 1799 ± 473 (*7*) SOD1: 1084 ± 322 (*5*) Not significant	WT: 1745 ± 132 (*5*) SOD1: 1901 ± 122 (*5*) Not significant	WT: 2184 ± 421 (*5*) SOD1: 2088 ± 298 (*5*) Not significant	WT: 1537 ± 127 (*6*) SOD1: 1376 ± 66 (*7*) Not significant
Basal arbor length (μm)	WT: 1122 ± 184 (*7*) SOD1: 765 ± 188 (*5*) Not significant	WT: 1199 ± 183 (*5*) SOD1: 2157 ± 258 (*5*) Not significant	WT: 2240 ± 485 (*5*) SOD1: 1783 ± 310 (*5*) Not significant	WT: 1653 ± 258 (*6*) SOD1: 1594 ± 360 (*7*) Not significant
Mean basal tree length (μm)	WT: 367 ± 52 (*7*) SOD1: 265 ± 70 (*5*) Not significant	WT: 353 ± 39 (*5*) SOD1: 553 ± 76 (*5*) Not significant	WT: 600 ± 109 (*5*) SOD1: 632 ± 152 (*5*) Not significant	WT: 598 ± 103 (*6*) SOD1: 486 ± 85 (*7*) Not significant
Apical reach (μm)	WT: 439 ± 39 (*7*) SOD1: 492 ± 21 (*5*) Not significant	WT: 557 ± 39 (*5*) SOD1: 562 ± 21 (*5*) Not significant	WT: 544 ± 55 (*5*) SOD1: 568 ± 85 (*5*) Not significant	WT: 608 ± 33 (*6*) SOD1: 626 ± 21 (*7*) Not significant
Basal reach (μm)	WT: 130 ± 18 (*7*) SOD1: 107 ± 17 (*5*) Not significant	WT: 180 ± 28 (*5*) SOD1: 204 ± 34 (*5*) Not significant	WT: 172 ± 10 (*5*) SOD1: 213 ± 7 (*5*) Not significant	WT: 184 ± 21 (*6*) SOD1: 181 ± 16 (*7*) Not significant
Apical ramifications	WT: 6.1 ± 0.6 (*7*) SOD1: 5.1 ± 0.6 (*5*) Not significant	WT: 7 ± 0.6 (*5*) SOD1: 5.4 ± 0.5 (*5*) Not significant	WT: 9.4 ± 1.6 (*5*) SOD1: 7.4 ± 0.6 (*5*) Not significant	WT: 5.4 ± 0.5 (*6*) SOD1: 5.3 ± 0.5 (*7*) Not significant
Basal ramifications	WT: 5.3 ± 1.1 (*7*) SOD1: 4.7 ± 1 (*5*) Not significant	WT: 4.9 ± 0.7 (*5*) SOD1: 4.6 ± 0.4 (*5*) Not significant	WT: 5.1 ± 0.4 (*5*) SOD1: 4.9 ± 0.6 (*5*) Not significant	WT: 5.9 ± 0.4 (*6*) SOD1: 5 ± 0.3 (*7*) Not significant
Apical spine density per 100 μm	WT: 75.5 ± 14.4 (*7*) SOD1: 78.5 ± 4.1 (*5*) Not significant	WT: 44.1 ± 5.1 (*5*) SOD1: 45 ± 4.2 (*5*) Not significant	WT: 36 ± 7.3 (*5*) SOD1: 37.6 ± 6.6 (*5*) Not significant	WT: 52.1 ± 9.5 (*6*) SOD1: 52.4 ± 4.6 (*7*) Not significant
Basal spine density per 100 μm	WT: 70.6 ± 8.3 (*7*) SOD1: 70 ± 11.1 (*5*) Not significant	WT: 47 ± 4 (*5*) SOD1: 38.6 ± 4.4 (*5*) Not significant	WT: 30.2 ± 5.1 (*5*) SOD1: 34.8 ± 10.3 (*5*) Not significant	WT: 42 ± 2.2 (*6*) SOD1: 52.1 ± 5.5 (*7*) Not significant

All data presented as mean ± SEM. All analyses were unpaired two-way ANOVAs for age and genotype, comparing soma volume (Age: P = 0.0077^**^; Genotype: P = 0.5572), total dendritic arbor length (Age: P = 0.0081^**^; Genotype: P = 0.6043), apical dendritic arbor length (Age: P = 0.0791; Genotype: P = 0.3395), basal dendritic arbor length (Age: P = 0.0094^**^; Genotype: P = 0.9479), mean basal dendritic tree length (Age: P = 0.0203^*^; Genotype: P = 0.9492), apical dendritic reach (Age: P = 0.0221^*^; Genotype: P = 0.1927), basal dendritic reach (Age: P = 0.0024^*^; Genotype: P = 0.3040), apical branch ramifications (Age: P = 0.0058^**^; Genotype: P = 0.1049), basal branch ramifications (Age: P = 0.7941; Genotype: P = 0.1781), apical dendritic spine density per 100 μm (Age: P = 0.0002^***^; Genotype: P = 0.8124), and basal dendritic spine density per 100 μm (Age: P < 0.0001^****^; Genotype: P = 0.7623). Bonferroni post-tests were used to compare the effect of genotype at each age group, with the adjusted P-value (adj. P) reported in the table for parameters that had significant genotype effects. Each animal used (n) contains mean values from multiple individual neurons, specifically P8-15 (WT: 11 neurons; SOD1: 10 neurons), P28-35 (WT: 10 neurons; SOD1: 5 neurons), P65-75 (WT: 8 neurons; SOD1: 7 neurons), and P120 (WT: 16 neurons; SOD1: 14 neurons).

### Brainstem SOD1 XII MNs show an early increase in dendritic arbor length, followed by a later decrease in dendritic arbor length

**Figure 2:**
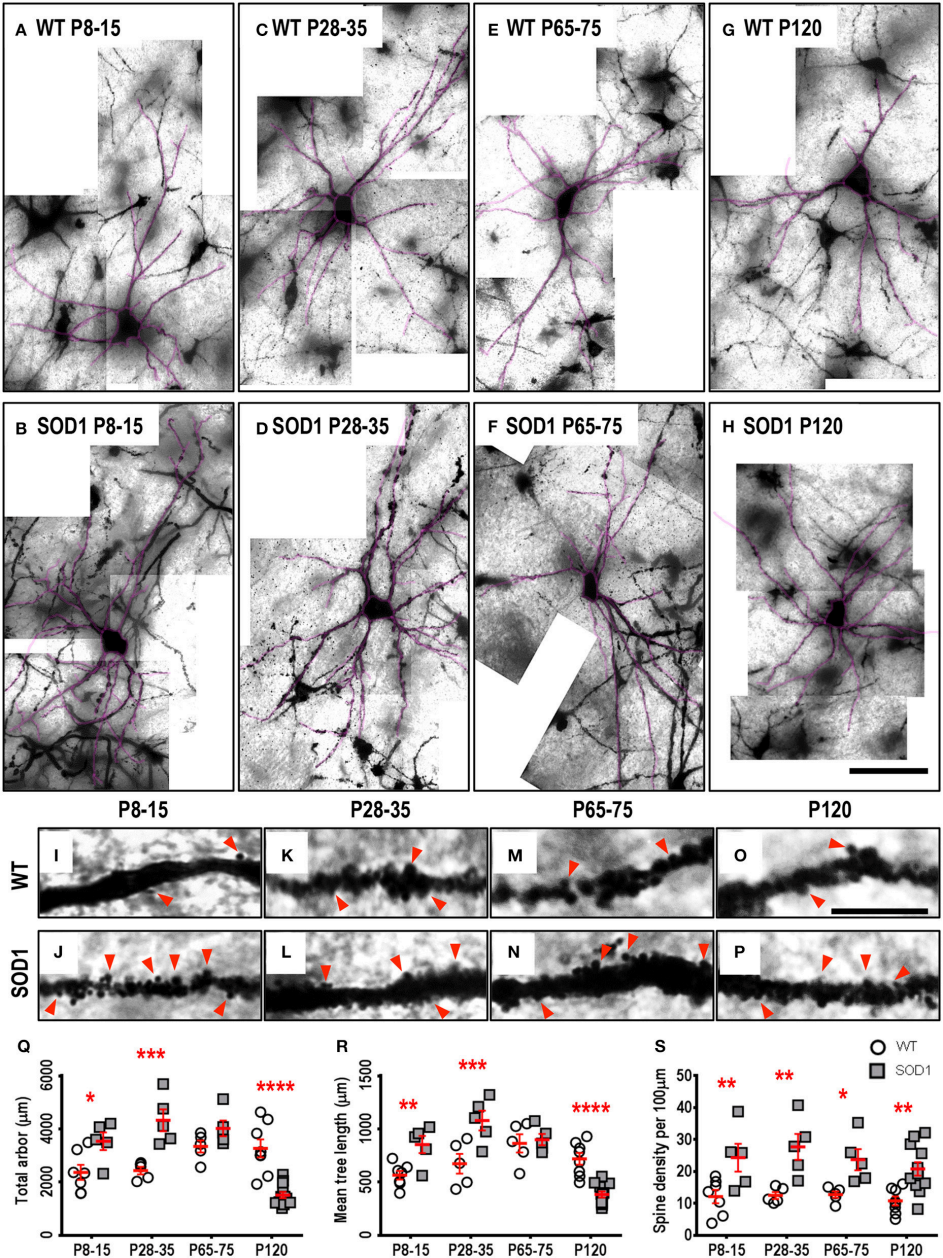
Altered dendritic arbors and increased spine density of SOD1 XII MNs commencing from P8-15 compared to WT controls. Images show mosaics of XII MNs from P8-15, P28-35, P65-75, and P120 WT (**A**,**C**,**E**,**G**) and SOD1 (**B**,**D**,**F**,**H**) mice brainstem. Superimposed in purple are dendritic tracings from the XII MN in order to illustrate more clearly the dendritic arbor size differences. High-magnification images of dendrites from P8-15, P28-35, P65-75, and P120 WT (**I**,**K**,**M**,**O**) and SOD1 (**J**,**L**,**N**,**P**) XII MNs are also shown, with examples of some of the dendritic spines identified using red arrows. Note that this is representative of the spiny structures and not an exhaustive identification of all quantified spines on these images. **(Q)** shows a scatterplot quantifying significantly increased total dendritic length (μm) of SOD1 XII MNs (gray squared) at P8-15 and 28-35 and significant decreases at P120 compared to WT controls (white circles). **(R)** shows a scatterplot quantifying significantly increased mean tree dendritic length (μm) of SOD1 XII MNs (gray squared) at P8-15 and 28-35 and significant decreases at P120 compared to WT controls (white circles). **(S)** shows a scatterplot quantifying significantly increased dendritic spine density per 100 μm of SOD1 XII MNs (gray squared) compared to WT controls (white circles) at P8-15, P28-35, P65-75, and P120. All data mean ± SEM, with two-way ANOVAs followed by Bonferroni post-tests, ^*^P < 0.05, ^**^P < 0.01, ^***^P < 0.001, and ^****^P < 0.0001. *n* = 7, 5, 5, and 8 for WT P8-15, P28-35, P65-75, and P120 respectively. *n* = 6, 5, 5, and 12 for SOD1 P8-15, P28-35, P65-75, and P120 respectively. Scale bar: **(A–H)**, 100 μm. Dendritic frame width **(I–P)**, 15 μm.

Approximately one third of ALS patients show bulbar deficits as their first symptom, evident as impaired speech or swallowing that can lead to malnutrition, choking, and death (Fujimura-Kiyono et al., 2011). Synaptic and intrinsic hyper-excitability of XII MNs occurs in neonatal SOD1 mice (van Zundert et al., 2008), and oral motor deficits also occur in ALS rodent models (Smittkamp et al., 2008). At P8-15 in SOD1 XII MNs, the total arbor length was increased by 78%, compared to WT MNs (*P* = 0.015^*^, Table 3, Figure 2Q). This increase persisted at a lower level of 20% at P28-35 in SOD1 XII MNs, compared to WT MNs (*P* = 0.021^*^, Table 3, Figure 2Q). By P65-75, the total arbor length of SOD1 XII MNs was the same as WT controls (*P* = 0.48, Table 3, Figure 2Q). By P120, total arbor length of SOD1 XII MNs was reduced by 54%, compared to WT XII MNs (*P* < 0.0001^****^, Table 3, Figure 2Q). This pattern of changes was repeated for the mean dendritic tree length. Compared to WT XII MNs, the mean tree length of SOD1 XII MNs SOD1 XII MNs was increased by 51% at P8-15 increased (*P* < 0.009^**^, Table 3, Figure 2R), increased by 60% at P28-35 (*P* < 0.0005^***^, Table 3, Figure 2R), not different at P65-75 (Table 3, Fig. 2 Q), and decreased by 46% decrease at P120 (*P* < 0.0001^****^, Table 3, Figure 2R). The soma volume of XII MNs was unchanged between genotypes at all ages studied (Table 3). There was also no significant difference in the dendritic reach or dendritic ramifications of XII MNs of SOD1 and WT mice for any age studied (Table 3).

**Table 3:** Morphometric dendritic and dendritic spine parameters of XII MNs within the brainstem.

**Region**	**P8-15 (*n*) Neonatal**	**P28-35 (*n*) Pre-symptomatic**	**P65-75 (*n*) Symptom Onset**	**P120 (*n*) Mid-disease**
Soma volume (μm^3^)	WT: 22920 ± 4672 (*7*) SOD1: 35226 ± 5774 (*5*) Not significant	WT: 40267 ± 5000 (*5*) SOD1: 39171 ± 4447 (*5*) Not significant	WT: 34463 ± 3246 (*5*) SOD1: 52278 ± 4821 (*5*) Not significant	WT: 37516 ± 6432 (*8*) SOD1: 16578 ± 1934 (*12*) Not significant
Total arbor length (μm)	WT: 2369 ± 284 (*7*) SOD1: 3537 ± 332 (*5*) Adj. *P* = 0.0208^*^	WT: 2437 ± 141 (*5*) SOD1: 4327 ± 409 (*5*) Adj. *P* = 0.0003^***^	WT: 3343 ± 226 (*5*) SOD1: 4023 ± 295 (*5*) Adj. *P* = 0.4814	WT: 3269 ± 338 (*8*) SOD1: 1519 ± 115 (*12*) Adj. *P* < 0.0001^****^
Mean tree length (μm)	WT: 567 ± 41 (*7*) SOD1: 854 ± 80 (*5*) Adj. *P* = 0.0094^**^	WT: 673 ± 93 (*5*) SOD1: 1079 ± 93 (*5*) Adj. *P* = 0.0005^***^	WT: 864 ± 86 (*5*) SOD1: 900 ± 53 (*5*) Adj. *P* > 0.9999	WT: 719 ± 56 (*8*) SOD1: 385 ± 28 (*12*) Adj. *P* < 0.0001^****^
Dendritic reach (μm)	WT: 339 ± 19 (*7*) SOD1: 491 ± 51 (*5*) Not significant	WT: 436 ± 40 (*5*) SOD1: 605 ± 6 (*5*) Not significant	WT: 575 ± 35 (*5*) SOD1: 658 ± 42 (*5*) Not significant	WT: 540 ± 53 (*8*) SOD1: 338 ± 17 (*12*) Not significant
Dendritic ramifications	WT: 4.7 ± 0.5 (*7*) SOD1: 5.3 ± 0.1 (*5*) Not significant	WT: 3.9 ± 0.3 (*5*) SOD1: 5.5 ± 0.2 (*5*) Not significant	WT: 4.7 ± 0.3 (*5*) SOD1: 5.2 ± 0.4 (*5*) Not significant	WT: 5.6 ± 0.2 (*8*) SOD1: 4.2 ± 0.4 (*12*) Not significant
Dendritic spine density per 100 μm	WT: 12.1 ± 2.1 (*7*) SOD1: 24.3 ± 4.3 (*5*) Adj. *P* = 0.0099^**^	WT: 12.5 ± 1.1 (*5*) SOD1: 27.7 ± 4 (*5*) Adj. *P* = 0.0023^**^	WT: 12.8 ± 1 (*5*) SOD1: 23.7 ± 3.3 (*5*) Adj. *P* = 0.0419^*^	WT: 10.8 ± 1.4 (*8*) SOD1: 20.8 ± 2.1 (*12*) Adj. *P* = 0.0051^**^

All data presented as mean ± SEM. All analyses were unpaired two-way ANOVAs for age and genotype, comparing soma volume (Age: P = 0.0019^**^; Genotype: P = 0.5533), total dendritic arbor length (Age: P < 0.0001^****^; Genotype: P = 0.0154^*^), mean dendritic tree length (Age: P < 0.0001^****^; Genotype: P = 0.0313^*^), dendritic reach (Age: P < 0.0001^****^; Genotype: P = 0.0758), dendritic ramifications (Age: P = 0.8886; Genotype: P = 0.2070), and dendritic spine density per 100 μm (Age: P = 0.3741; Genotype: P < 0.0001^****^). Bonferroni post-tests were used to compare the effect of genotype at each age group, with the adjusted P-value (adj. P) reported in the table for parameters that had significant genotype effects. Each animal used (n) contains mean values from a minimum of 2 individual neurons, specifically P8-15 (WT: 21 neurons; SOD1: 22 neurons), P28-35 (WT: 12 neurons; SOD1: 18 neurons), P65-75 (WT: 12 neurons; SOD1: 11 neurons), and P120 (WT: 18 neurons; SOD1: 27 neurons).

### Brainstem SOD1 XII MNs show increased dendritic spine density from P8-15 until P120

The dendritic spine is the postsynaptic compartment for excitatory synapses (Harris, 1999), and is present on the dendrites of XII MNs in neonatal WT mice (Kanjhan et al., 2015). Compared to WT littermates, the dendritic spine density of XII MNs from SOD1 mice was increased at all ages (Table 3, Figure 2). At P8-15 in SOD1 XII MNs, SOD1 XII MN spine density was increased by 101% (*P* = 0.0099^**^, Table 3, Figures 2I,J,S); at P28-35, spine density was increased by 122% (*P* = 0.0023^**^, Table 3, Figures 2K,L,S); at P65-75, spine density was increased by 85% (*P* = 0.042^*^, Table 3, Figures 2M,N,S); and at P120 (*P* = 0.0051^**^, Table 3, Figures 2O,P,S), spine density was increased by 93%, compared to WT controls at the same ages (Table 3, Figure 2S).

### Lumbar spinal cord MNs of SOD1 mice show reductions in dendritic length from P28-35 onward

**Figure 3:**
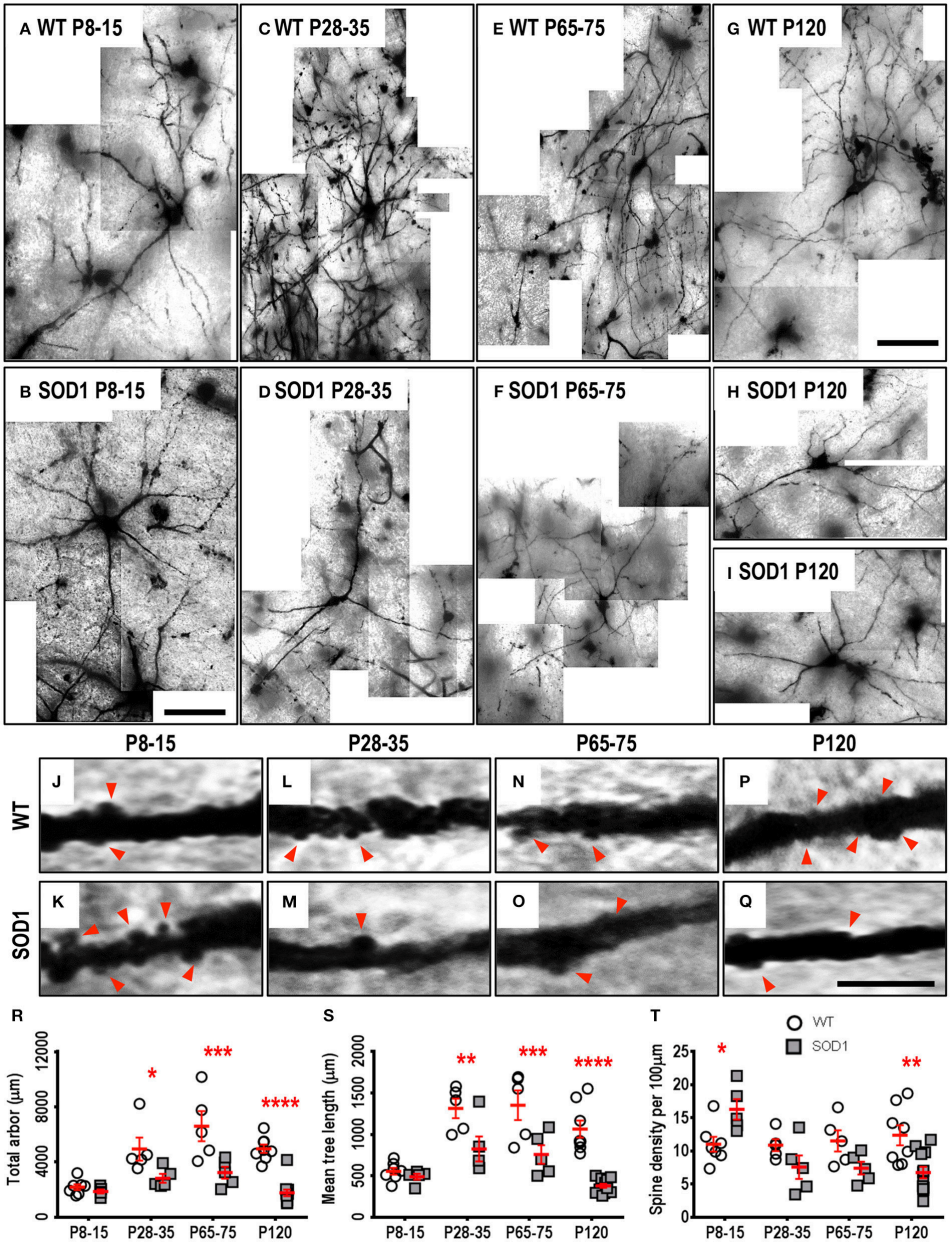
Decreased dendritic arbors of SOD1 lumbar MNs from P28-35 onward, and altered spine density from P8-15 onward, compared to WT controls. Images show mosaics of lumbar MNs from P8-15, P28-35, P65-75, and P120 WT (**A**,**C**,**E**,**G**) and SOD1 (**B**,**D**,**F**,**H**,**I**) spinal cords. High-magnification images of dendrites from P8-15, P28-35, P65-75, and P120 WT (**J**,**L**,**N**,**P**) and SOD1 (**K**,**M**,**O**,**Q**) lumbar MNs are also shown, with examples of some of the dendritic spines identified using red arrows. Note that this is representative of the spiny structures and not an exhaustive identification of all quantified spines on these images. **(R)** shows a scatterplot quantifying significantly decreased total dendritic length (μm) of SOD1 lumbar MNs (gray squared) compared to WT controls (white circles) at P28-35, P65-75, and P120. **(S)** shows a scatterplot quantifying significantly decreased mean tree dendritic length (μm) of SOD1 lumbar MNs (gray squared) compared to WT controls (white circles) at P28-35, P65-75, and P120. **(T)** shows a scatterplot quantifying significantly increased dendritic spine density per 100 μm of SOD1 lumbar MNs (gray squared) at P8-16 and significant decreases at P120 compared to WT controls (white circles). All data mean ± SEM, with two-way ANOVAs followed by Bonferroni post-tests, ^*^P < 0.05, ^**^P < 0.01, ^***^P < 0.001, and ^****^P < 0.0001. *n* = 7, 5, 5, and 8 for WT P8-15, P28-35, P65-75, and P120 respectively. *n* = 6, 5, 5, and 12 for SOD1 P8-15, P28-35, P65-75, and P120 respectively. Scale bar: **(A**,**B)**, 75 μm. **(C**–**I)**, 150 μm. Dendritic frame width, 20 μm **(J–Q)**.

As respiratory MNs (which include XII MNs) tend to degenerate later than spinal cord MNs innervating limb muscles (Haenggeli and Kato, 2002; Ngo et al., 2012; Lee et al., 2013), we determined whether the dendritic changes in XII MNs were reiterated in hindlimb MNs of the lumbar spinal cord MNs of SOD1, compared to WT mice. By contrast to the early increase in dendritic length seen in XII MNs from neonatal or pre-symptomatic SOD1 mice, the dendritic arbors of SOD1 lumbar MNs did not increase, but were decreased from P28-35 onward, compared to WT controls (Figure 3). Compared to WT controls, the total arbor length of SOD1 lumbar MNs was unchanged at P8-15 (Table 4, Figure 3R), decreased by 43% at P28-35 (*P* = 0.023^*^, Table 4, Figure 3R), decreased by 51% at P65-75 (*P* = 0.0001^***^, Table 4, Figure 3R), and decreased by 64% at P120 (*P* < 0.0001^****^, Table 4, Figure 3R). This pattern was repeated for mean dendritic tree lengths of SOD1 lumbar MNs, compared to WT controls, being unchanged at P8-15 (Table 4, Figure 3S), decreased by 37% at P28-35 (*P* = 0.0073^**^, Table 4, Figure 3S), decreased by 44% at P65-75 (*P* = 0.0009^***^, Table 4, Figure 3S), and decreased by 65% at P120 (*P* < 0.0001^****^, Table 4, Figure 3S). A similar pattern was seen for changes in dendritic reach, which was unchanged for P8-15 SOD1 lumbar MNs (*P* > 0.9999, Table 4), decreased at P28-35 by 31% (*P* = 0.033^*^, Table 4), decreased at P65-75 by 32% (*P* = 0.0032^**^, Table 4), and further decreased at P120 by 46% (*P* < 0.0001^****^, Table 4), compared to WT controls at the same ages.

**Table 4:** Morphometric dendritic and dendritic spine parameters of MNs in the lumbar spinal cord.

**Region**	**P8-15 (*n*) Neonatal**	**P28-35 (*n*) Pre-symptomatic**	**P65-75 (*n*) Symptom onset**	**P120 (*n*) Mid-disease**
Soma volume (μm^3^)	WT: 17937 ±3476 (*7*) SOD1: 16895 ± 1966 (*5*) Adj. *P* > 0.9999	WT: 46512 ± 3182 (*5*) SOD1: 36391 ± 2769 (*5*) Adj. *P* = 0.1530	WT: 38309 ± 5299 (*5*) SOD1: 43454 ± 3571 (*5*) Adj. *P* = 0.9999	WT: 31198 ± 2331 (*8*) SOD1: 13659 ± 1816 (*12*) Adj. *P* < 0.0001^****^
Total arbor length (μm)	WT: 2195 ± 205 (*7*) SOD1: 1873 ± 149 (*5*) Adj. *P* > 0.9999	WT: 4945 ± 836 (*5*) SOD1: 2828 ± 314 (*5*) Adj. *P* = 0.0226^*^	WT: 6604 ± 1090 (*5*) SOD1: 3231 ± 427 (*5*) Adj. *P* = 0.0001^***^	WT: 4971 ± 299 (*8*) SOD1: 1778 ± 240 (*12*) Adj. *P* < 0.0001^****^
Mean tree length (μm)	WT: 560 ± 41 (*7*) SOD1: 490 ± 36 (*5*) Adj. *P* > 0.9999	WT: 1315 ± 118 (*5*) SOD1: 826 ± 151 (*5*) Adj. *P* = 0.0023^**^	WT: 1353 ± 179 (*5*) SOD1: 758 ± 113 (*5*) Adj. *P* = 0.0009^***^	WT: 1073 ± 102 (*8*) SOD1: 380 ± 24 (*12*) Adj. *P* < 0.0001^****^
Dendritic reach (μm)	WT: 351 ± 40 (*7*) SOD1: 330 ± 32 (*5*) Adj. *P* > 0.9999	WT: 595 ± 58 (*5*) SOD1: 413 ± 23 (*5*) Adj. *P* = 0.0334^*^	WT: 756 ± 83 (*5*) SOD1: 517 ± 55 (*5*) Adj. *P* = 0.0032^**^	WT: 562 ± 32 (*8*) SOD1: 302 ± 21 (*12*) Adj. *P* = 0.0001^****^
Dendritic ramifications	WT: 4.7 ± 0.4 (*7*) SOD1: 4.3 ± 0.2 (*5*) Adj. *P* > 0.9999	WT: 5.2 ± 0.6 (*5*) SOD1: 4.6 ± 0.1 (*5*) Adj. *P* > 0.9999	WT: 7.2 ± 0.6 (*5*) SOD1: 5.0 ± 0.3 (*5*) Adj. *P* = 0.0176^*^	WT: 7.4 ± 0.7 (*8*) SOD1: 4.3 ± 0.2 (*12*) Adj. *P* = 0.0001^****^
Dendritic spine density per 100 μm	WT: 11.0 ± 1.1 (*7*) SOD1: 16.3 ± 1.5 (*5*) Adj. *P* = 0.0374^*^	WT: 10.8 ± 0.9 (*5*) SOD1: 7.6 ± 1.8 (*5*) Adj. *P* = 0.4785	WT: 11.5 ± 1.6 (*5*) SOD1: 7.4 ± 1 (*5*) Adj. *P* = 0.2205	WT: 12.4 ± 1.5 (*8*) SOD1: 6.8 ± 0.9 (*12*) Adj. *P* = 0.0021^**^

All data are mean ± SEM. All analyses were unpaired two-way ANOVAs for age and genotype, comparing soma volume (Age: P < 0.0001^****^; Genotype: P = 0.0097^**^), total dendritic arbor length (Age: P < 0.0001^****^; Genotype: P < 0.0001^****^), mean dendritic tree length (Age: P < 0.0001^****^; Genotype: P < 0.0001^****^), dendritic reach (Age: P < 0.0001^****^; Genotype: P < 0.0001^****^), dendritic ramifications (Age: P = 0.0036^**^; Genotype: P = 0.0001^****^), and dendritic spine density per 100 μm (Age: P = 0.0051^**^; Genotype: P < 0.0481^*^). Bonferroni post-tests were used to compare the effect of genotype at each age group, with the adjusted P-value (adj. P) reported in the table for parameters that had significant genotype effects. Each animal used (n) contains mean values from a minimum of 2 individual neurons, specifically P8-15 (WT: 25 neurons; SOD1: 13 neurons), P28-35 (WT: 12 neurons; SOD1: 18 neurons), P65-75 (WT: 13 neurons; SOD1: 19 neurons), and P120 (WT: 30 neurons; SOD1: 39 neurons).

The branching of SOD1 lumbar MN dendrites was also reduced at later disease stages. At P8-15 and P28-35, the dendritic ramifications of SOD1 lumbar MNs were unchanged, compared to WT controls (Table 4), while dendritic ramifications decreased by 31% at P65-75 (*P* = 0.018^*^, Table 4), and then declined further to 42% at P120 in SOD1 lumbar MNs (*P* < 0.0001^****^, Table 4), compared to WT controls at the same ages.

Finally, SOD1 lumbar MN soma volume was unchanged for the three earliest age groups (Table 4). However, by P120, the soma volume of SOD1 lumbar MNs, was reduced by 56%, compared to WT controls (*P* < 0.0001^****^, Table 4).

### Dendritic spine density of SOD1 lumbar MNs is increased at P8-15, followed by a decrease in dendritic spine density by P120

We next determined whether reduced dendritic arbors of SOD1 lumbar MNs were associated with decreases in dendritic spine density, similar to changes in motor cortex and medial prefrontal cortex pyramidal neurons of SOD1 mice (Sgobio et al., 2008; Jara et al., 2012; Fogarty et al., 2015, 2016c) or whether dendritic spines were increased, as for XII MNs. Compared to WT controls, dendritic spine density of SOD1 lumbar MNs increased by 48% at P8-15 (*P* = 0.037^*^, Table 4, Figures 3J,K,T), then returned to control levels at P28-35 and P65-75 (Table 4, Figures 3L–O,T), and decreased by 45% at P120 compared to WT controls by (*P* = 0.0021^**^, Table 4, Figures 3P,Q,T). Thus, both XII MNs and lumbar MNs had increased dendritic spine densities at P8-16, compared to WT controls. However, while spine density in lumbar MNs then returned to control levels at P28-25 and P65-75, and then decreased at P120, spine density remained elevated in XII MNs at all ages studied. Elevated spine density could be thus interpreted as a neuroprotective response, as lumbar MNs are beginning to die by P30-35 in SOD1 mice (Ngo et al., 2012; Vinsant et al., 2013), while XII MN loss occurs later, at P90-100 (Haenggeli and Kato, 2002).

### Brainstem IV SOD1 MNs show no dendritic or spine differences compared to WT controls at any age studied

Some motor neuron pools are relatively resistant to death in ALS, such as those innervating the external muscles of the eye (Ferrucci et al., 2010). We therefore assessed the dendritic arbor and dendritic spine densities of trochlear (IV) MNs in the brainstem. There was no morphological changes in soma volume, total arbor length, mean tree length, dendritic reach, dendritic ramifications, or dendritic spine density of IV MNs from SOD1 mice, compared to WT IV MNS, for any age studied (Table 5). This lack of morphological change in IV MNs of SOD1 mice fits with the relative sparing of IV MNs in this model (Ferrucci et al., 2010).

**Table 5:** Morphometric dendritic and dendritic spine parameters of IV MNs within the brainstem.

**Region**	**P8-15 (*n*) Neonatal**	**P28-35 (*n*) Pre-symptomatic**	**P65-75 (*n*) Symptom onset**	**P120 (*n*) Mid-disease**
Soma volume (μm^3^)	WT: 21787 ± 2174 (*7*) SOD1: 18415 ± 2391 (*5*) Not significant	WT: 24493 ± 2000 (*5*) SOD1: 21604 ± 2394 (*5*) Not significant	WT: 29634 ± 3835 (*5*) SOD1: 24497 ± 4541 (*5*) Not significant	WT: 26868 ± 1150 (*8*) SOD1: 25378 ± 2525 (*12*) Not significant
Total arbor length (μm)	WT: 1370 ± 156 (*7*) SOD1: 1119 ± 129 (*5*) Not significant	WT: 1495 ± 181 (*5*) SOD1: 1563 ± 130 (*5*) Not significant	WT: 2053 ± 230 (*5*) SOD1: 1714 ± 83 (*5*) Not significant	WT: 1728 ± 116 (*8*) SOD1: 1686 ± 150 (*12*) Not significant
Mean tree length (μm)	WT: 371 ± 44 (*7*) SOD1: 275 ± 17 (*5*) Not significant	WT: 342 ± 53 (*5*) SOD1: 392 ± 62 (*5*) Not significant	WT: 529 ± 55 (*5*) SOD1: 446 ± 50 (*5*) Not significant	WT: 370 ± 24 (*8*) SOD1: 472 ± 45 (*12*) Not significant
Dendritic reach (μm)	WT: 157 ± 12 (*7*) SOD1: 133 ± 9 (*5*) Not significant	WT: 170 ± 7 (*5*) SOD1: 194 ± 8 (*5*) Not significant	WT: 270 ± 14 (*5*) SOD1: 232 ± 7 (*5*) Not significant	WT: 215 ± 10 (*8*) SOD1: 202 ± 13 (*12*) Not significant
Dendritic ramifications	WT: 3.7 ± 0.2 (*7*) SOD1: 3.8 ± 0.3 (*5*) Not significant	WT: 3.4 ± 0.5 (*5*) SOD1: 4.5 ± 0.5 (*5*) Not significant	WT: 3.2 ± 0.3 (*5*) SOD1: 3.2 ± 0.4 (*5*) Not significant	WT: 3.6 ± 0.1 (*8*) SOD1: 3.9 ± 0.3 (*12*) Not significant
Dendritic spine density per 100 μm	WT: 10.6 ± 3.6 (*7*) SOD1: 13.4 ± 5 (*5*) Not significant	WT: 10.6 ± 1.5 (*5*) SOD1: 15 ± 3.2 (*5*) Not significant	WT: 13 ± 1.5 (*5*) SOD1: 13.8 ± 1.5 (*5*) Not significant	WT: 8.6 ± 1.9 (*8*) SOD1: 9.4 ± 1.5 (*12*) Not significant

All data presented as mean ± SEM. All analyses were unpaired two-way ANOVAs for age and, comparing soma volume (Age: P = 0.0732; Genotype: P = 0.1188), total dendritic arbor length (Age: P = 0.0043^**^; Genotype: P = 0.3305), mean dendritic tree length (Age: P = 0.0183^*^; Genotype: P = 0.8501), dendritic reach (Age: P < 0.0001^****^; Genotype: P = 0.1711), dendritic ramifications (Age: P = 0.2077; Genotype: P = 0.1418), and dendritic spine density per 100 μm (Age: P = 0.2580; Genotype: P = 0.2489). Bonferroni post-tests were used to compare the effect of genotype at each age group, with the adjusted P-value (adj. P) reported in the table for parameters that had significant genotype effects. Each animal used (n) contains mean values from multiple neurons, specifically P8-15 (WT: 9 neurons; SOD1: 6 neurons), P28-35 (WT: 6 neurons; SOD1: 8 neurons), P65-75 (WT: 6 neurons; SOD1: 6 neurons), and P120 (WT: 11 neurons; SOD1: 15 neurons).

### Vacuolation occurs in XII MNs and lumbar MNs from P65-76 onward, but is not present in IV MNs

**Figure 4:**
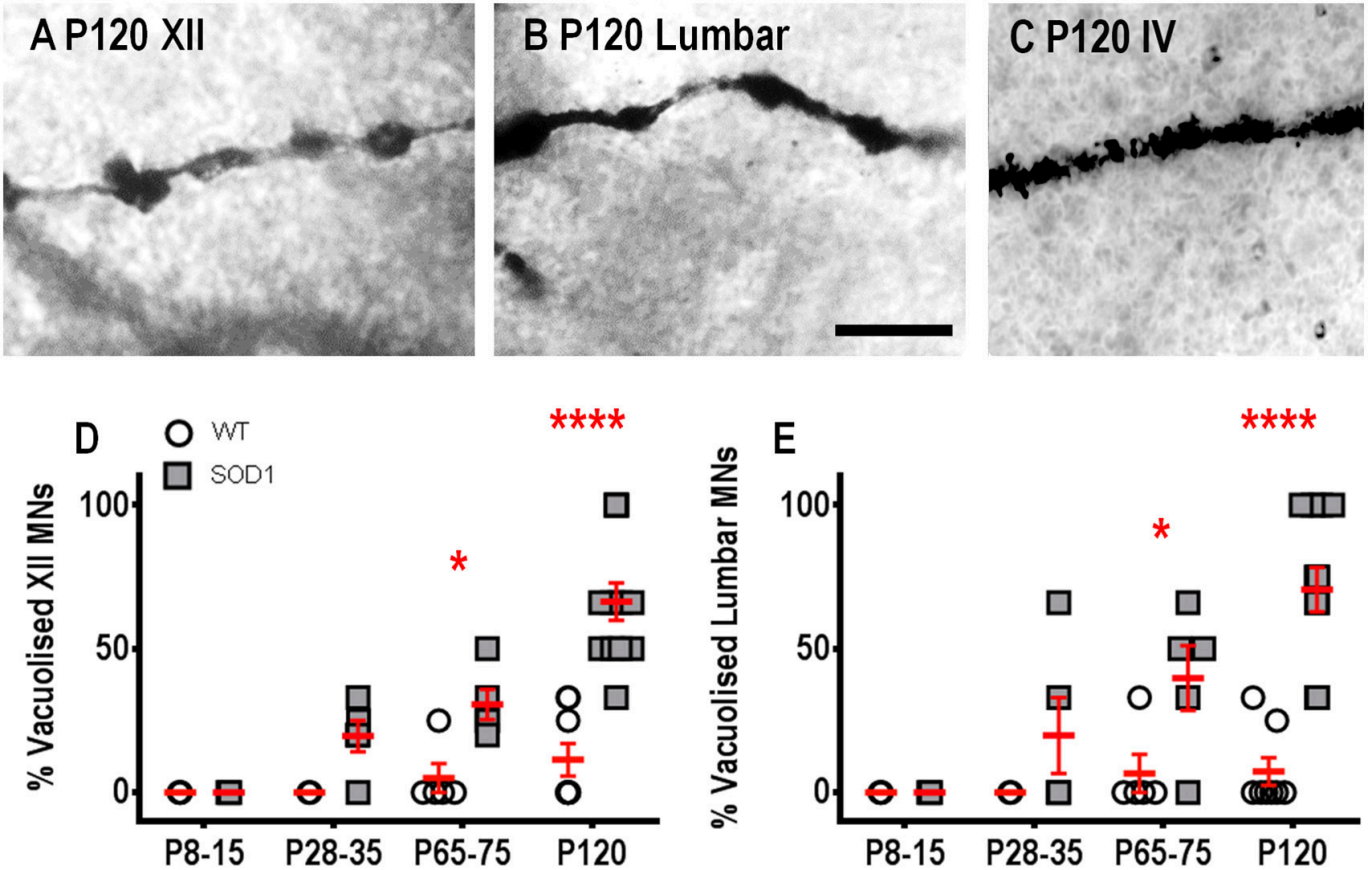
Increased proportion of vacuolated dendrites from SOD1 XII and lumbar MNs from P65-75 onward, compared to WT controls. Images show “beads on a string” appearance of dendritic vacuoles from P120 SOD1 XII MNs **(A)** and lumbar MNs **(B)**. High-magnification images of dendrites from P120 SOD1 IV MNs **(C)** shows lack of hollowing and vacuoles in this neuronal population. **(D)** shows a scatterplot quantifying increased percentages of vacuolated SOD1 XII MNs (gray squared) compared to WT controls (white circles) at P65-75 and P120. **(E)** shows a scatterplot quantifying increased percentages of vacuolated SOD1 lumbar MNs (gray squared) compared to WT controls (white circles) at P65-75 and P120. All data mean ± SEM, with two-way ANOVAs followed by Bonferroni post-tests, ^*^P < 0.05 and ^*****^P < 0.0001. *n* = 7, 5, 5, and 8 for WT P8-15, P28-35, P65-75, and P120 respectively. *n* = 6, 5, 5, and 12 for SOD1 P8-15, P28-35, P65-75, and P120 respectively. Scale bar: **(A,B)**, 25 μm.

Dendritic vacuoles, derived from degenerating mitochondria, are prevalent in lumbar MNs from P60 onward in SOD1 mice (Kong and Xu, 1998; Vinsant et al., 2013) and in the dendrites of human Betz cells in ALS post mortem tissue (Genç et al., 2017). Similar to other previous studies of vacuolization (Rafols and Fox, 1979; Goldstein et al., 1983), we found abundant dendritic vacuolation in XII MNs and lumbar MNs, at both P65-75 and P120, compared to WT MNs, quantified on a presence/absence basis (Figure 4). For the two earlier age groups, there were no differences in the percentage of XII MNs with vacuolization (Figure 4D); however, by P65-75, the percentage of MNs with vacuolization increased by ~6-fold, compared to WT controls (*P* = 0.028^*^, Figure 4D), and this increase persisted at P120 (*P* < 0.0001^****^ Figure 4D). Similarly, at P8-15 and P28-35, lumbar MNs in SOD1 mice did not show an increase in vacuolization, compared to WT controls (Figure 4E), but, at P65-75, there was a ~6-fold increase in the percentage of MNs with vacuolization (*P* = 0.034^*^, Figure 4E) and by P120, this became a 10-fold increase in the percentage of lumbar MNs with vacuolization, (*P* < 0.0001^****^ Figure 4E). By contrast, IV MNs from SOD1 mice did not exhibit significant differences in vacuolization at any age studied, compared to WT controls (*P* = 0.37, Figure 4C).

## Discussion

There are no longitudinal studies of dendritic morphology and spine density of several subcortical and motor neuron populations in any rodent model of ALS. We present a number of new insights, showing that morphological changes occur in MSNs from the dorsal striatum, brainstem XII MNs, and lumbar MNs, but not in hippocampal CA1 pyramidal neurons, and brainstem IV MNs. The structural changes in brainstem and spinal cord MNs susceptible to death in ALS exhibited differing degrees of dendritic and dendritic spine plasticity, as well as different rates of change, which may reflect differential susceptibility to dendritic degeneration. In total, our morphological assessment of dendritic changes strengthens the idea that changes in dendritic structure and synaptic inputs are drivers of both motor and non-motor phenotypes in ALS. As glutamate is the most widespread CNS neurotransmitter, our results provide additional support for the hypothesis that glutamate excitotoxicity as one of the pathological factors in ALS.

The striatum participates in control of mood, language and behavior, all of which can be abnormal in ALS (Josephs et al., 2009). Changes in striatum volume are present in ALS (Bede et al., 2013), and TDP-43 positive inclusions are found in striatal neurons in ALS patients (Josephs et al., 2009; Brettschneider et al., 2013; Riku et al., 2016). In the dorsal striatum, we found that there was a decrease in dendritic spine density of MSNs of SOD1 mice, without any changes in dendritic length or structure. Our dendritic length measurements agree with previous morphological studies of striatal MSNs; however, our spine densities are lower (for both genotypes) than those derived from fluorescent 3-dimensional rendering methodology (Judson et al., 2010), suggesting that the Golgi-Cox impregnation may be underestimating spine density differences between genotypes. Excitotoxic cell death in the striatum is associated with spine loss in MSNs (Garcia et al., 2010), and is also postulated to be a mechanism in neurodegenerative diseases of adulthood, including Parkinson's and Huntington's disease (Cepeda et al., 2003; Chase et al., 2003; Plotkin and Surmeier, 2015).

SOD1 mice show enhanced learning behavior and hippocampal long term synaptic plasticity (Spalloni et al., 2006). Unexpectedly, hippocampal CA1 pyramidal neurons from SOD1 mice displayed no morphological differences to controls at any age studied. The two major sources of excitatory input to CA1 pyramidal neurons are the Schaeffer collaterals of CA3 neurons, which synapse on the proximal apical dendrite, and layer II/III pyramidal neurons of the entorhinal cortex, which synapse on distal apical dendrites (van Groen et al., 2003). We have previously reported that pyramidal neurons in layer II/III of the entorhinal cortex, which also are the main efferent target of CA1 pyramidal neurons (van Groen et al., 2003), show no significant morphological changes in SOD1 mice (Fogarty et al., 2016c). Further functional studies of synaptic transmission within this hippocampal-entorhinal circuit are needed to test whether this circuitry undergoes significant modification in the SOD1 mouse.

In ALS, loss of brainstem XII MNs innervating the tongue contribute to disease morbidity and mortality, through impairment or loss of swallowing and speech, malnutrition, choking, and decreased ability to maintain upper airway patency for ventilation (Connolly et al., 2015). In SOD1 ALS models, XII MNs appear to undergo cell death later and at lower levels in comparison to spinal MNs (Haenggeli and Kato, 2002; Ngo et al., 2012; Lee et al., 2013). Thus, our observations of significant increases in dendritic arbor length and dendritic spine density suggest that these changes may underpin an enhanced capacity of SOD1 XII MNs to stave off degeneration. Alternatively, increased arborization and increased dendritic spines in SOD1 XII MNs may be maladaptive, increasing the substrate for later glutamate-induced excitotoxicity.

We found that XII MNs from SOD1 mice showed increased dendritic arbor length, from a week after birth until disease onset, followed by dendritic retraction by mid-disease age. Dendritic spine density was also elevated, compared to WT controls, at all ages studied. These findings in a vulnerable lower motor neuron population stand in contrast to the early dendritic shrinkage and spine loss of upper MNs and other vulnerable lower MNs reported here and elsewhere (Hammer et al., 1979; Horoupian et al., 1984; Udaka et al., 1986; Kato et al., 1987; Jara et al., 2012; Fogarty et al., 2015, 2016c). Although loss of dendritic arbor and dendritic spines are seen as hallmarks of neurodegeneration (Hammer et al., 1979; Horoupian et al., 1984; Udaka et al., 1986; Kato et al., 1987), other conditions, such as autism, exhibit increased spine density (Hutsler and Zhang, 2010), thereby suggesting that changes in circuit activity may not always be associated with spine loss, particularly in XII MNs (Fogarty et al., 2016a, 2017). Alternatively, since XII MNs have reduced calcium-buffering capacity (Rothstein et al., 1990; von Lewinski et al., 2008; Jaiswal and Keller, 2009), increased spine density may help to protect XII MNs, as spines can serve to confine calcium transients to the spine head (Koester and Sakmann, 1998; Yuste et al., 1999; Burette et al., 2010; Kenyon et al., 2010; Sala and Segal, 2014). This is consistent with reports of increased glutamatergic neurotransmission (van Zundert et al., 2008; Fogarty et al., 2016a, 2017) and intracellular calcium concentration (Bogaert et al., 2010; Luebke et al., 2010; Bellingham, 2011; van Zundert et al., 2012; Sala and Segal, 2014) in animal models showing increased MN excitability and of ALS.

We also found reduction in the dendritic arbor length of lumbar spinal cord MNs, compared to WT controls, commencing from P28-35. Similar to XII MNs, SOD1 lumbar MNs showed higher spine density compared to WT lumbar MNS at neonatal ages (P8-15), however, by disease onset (P65-75) and later, there was spine loss compared to WT controls. It is notable that hindlimb lumbar MNs are lost as early as P35 in SOD1 mice (Ngo et al., 2012), when both dendritic arbor length and spine density significantly decrease below WT controls. Previous reports have identified dendritic length recession in SOD1 mice at late-embryonic ages and at P6-10 (Martin et al., 2013; Leroy et al., 2014). The dendritic lengths reported here are lower than those obtained by single neuron dye-filling methods (Martin et al., 2013; Leroy et al., 2014); however, the dendritic length and spine density quantification were comparable to past studies using Golgi staining methods (Ma and Vacca-Galloway, 1991; Bou-Flores et al., 2000). Neonatal spinal cord MN morphology has been studied intensely in an alternate SOD1 model, the G83R mutation, which also shows increased dendritic length, compared to WT controls, at P3-4, P4-P9, and P8-9 (Amendola et al., 2007; Amendola and Durand, 2008; Filipchuk and Durand, 2012); a caveat is that dendritic lengthening is associated with MN hypoexcitability in this model (Pambo-Pambo et al., 2009; Hadzipasic et al., 2014). By contrast, lumbar MNs in SOD1 mice are hyper-excitable at postnatal ages (Quinlan et al., 2011), but begin to show hypo-excitability by P40 (Delestree et al., 2014). Further study of the relationship between functional MN excitability and MN morphology (Fogarty et al., 2013) will be needed to determine whether dendritic changes are driven by a shift from hyper-activity to hypo-activity.

Brainstem MNs controlling the extrinsic eye muscles, and sacral MNs controlling bladder and bowel function are relatively resistant to death in in clinical ALS and SOD1 models (Ferrucci et al., 2010; Spalloni and Longone, 2015). We found that there were no significant changes in dendritic structure or spine density at all ages studied in IV MNs, which are one of these resilient MN populations. P*ost mortem* studies of ALS spinal cord show that another resilient MN population, Onuf's nucleus MNs, have shorter dendritic arbors (Takeda et al., 2014). Again, functional studies of IV MNs and MNs from Onuf's nucleus are needed, to determine whether these MNs show disease-related alteration in their activity.

There has been some preliminary evidence supporting disease-related changes in dendritic spine subtypes, classified conventionally as stubby, mushroom, and thin (Jones and Powell, 1969) in cortical neurons from a TDP-43 rodent model (Handley et al., 2017). Limitations of the light microscopic method (Harris et al., 1992; Arellano et al., 2007; Maiti et al., 2015) and evidence of a continuum of intermediate spine morphologies in past reports (Peters and Kaiserman-Abramof, 1970; Harris et al., 1992; Maiti et al., 2015) have precluded a robust and repeatable taxonomy of dendritic spines in material from the present study. Although there may be inherent limitations with differential impregnation of neurons with Golgi-Cox in case of neuro-degeneration, evidence from the literature demonstrates successful staining in animal models of neurodegenerative diseases (Ukabam, 1988; Spires et al., 2004) and human post-mortem tissue in cases of aging and Alzheimer's disease (Hanks and Flood, 1991).

In summary, we report the first longitudinal study of dendritic arbors, dendritic spines and dendritic vacuoles of several sub-cortical neuron populations, including multiple MN types, in a widely utilized mouse model of ALS. We describe dendritic reduction in susceptible neurons in SOD1 mice, compared to WT controls, commencing relatively early, at P8-15 or P28-35, ages prior to the emergence of symptoms, in spinal cord lumbar MNs, and at mid-disease stage in XII MNs. Spine density increases were present at all ages studied in SOD1 XII MNs, while spine density of lumbar MNs was increased neonatally, returning to control levels by P28-35 and decreasing compared to WT control by P120. Spine loss occurred in the absence of dendritic alteration in the MSNs of the striatum from P65-75, the age of disease symptom onset. Establishing whether dendritic spine loss in in ALS is a protective change, or a maladaptive change, (Kawashima et al., 1998) is an important avenue of investigation. The morphologic evidence we present here, along with past studies (Fogarty et al., 2015), suggests that spine loss largely precedes dendritic pathology in SOD1 mice. It will also be important to determine whether similar morphological changes occur in susceptible neurons in the more recently developed animal models of ALS-related fronto-temporal dementia that show inclusion bodies (Leigh et al., 1991; Katsuse and Dickson, 2005).

Characterizing changes in dendritic arbor and dendritic spines is useful to identify areas where neuronal alterations occur in ALS. Our results indicate early and persistent changes in the dendritic network of neuro-motor and extra-motor regions in the SOD1 model. The alterations in glutamatergic excitatory synaptic transmission previously reported at early ages in this mouse model (van Zundert et al., 2008; Fogarty et al., 2015; Saba et al., 2015) and which also occur in human induced pluripotent stem cells derived from ALS patients (Devlin et al., 2015) are consistent with our morphological results, lending support to the hypothesis of glutamate excitotoxicity. Our study provides a useful reference point for more detailed characterization of other models of ALS or fronto-temporal dementia that continue to emerge, in addition to being an important baseline for the study of possible therapies using functional or behavioral outcomes.

## Author contributions

EM, MF, NL, PN, and MB contributed to study design. EM and MF performed all experiments. MF, EM, NL, PN, and MB contributed to data analysis, writing, and editing of the manuscript.

### Conflict of interest statement

The authors declare that the research was conducted in the absence of any commercial or financial relationships that could be construed as a potential conflict of interest.
